# Association of elevated circulating monocyte-platelet aggregates with hypercoagulability in patients with nephrotic syndrome

**DOI:** 10.1186/s12959-024-00626-3

**Published:** 2024-06-28

**Authors:** Shi-Ping Na, Mei-Liang Ning, Ji-Fang Ma, Shuang Liang, Yan-Li Wang, Man-Shu Sui, Xiao-Fang Guo, Ying Ji, Hui-Yan Lyu, Xue-Ying Yuan, Yu-Shi Bao

**Affiliations:** 1 Department of Nephrology, First Affiliated Hospital of Harbin Medical University, Harbin Medical University, No.23 Youzheng Street, Harbin, 150001 China; 2 Department of Rheumatology, First Affiliated Hospital of Harbin Medical University, Harbin Medical University, Harbin, 150001 China

**Keywords:** Monocyte-platelet aggregates, Nephrotic syndrome, Hypercoagulable state, Thrombosis

## Abstract

**Background:**

Hypercoagulability emerges as a central pathological feature and clinical complication in nephrotic syndrome. Increased platelet activation and aggregability are closely related to hypercoagulability in nephrotic syndrome. Monocyte-platelet aggregates (MPAs) have been proposed to represent a robust biomarker of platelet activation. The aim of this study was to investigate levels of the circulating MPAs and MPAs with the different monocyte subsets to evaluate the association of MPAs with hypercoagulability in nephrotic syndrome.

**Methods:**

Thirty-two patients with nephrotic syndrome were enrolled. In addition, thirty-two healthy age and sex matched adult volunteers served as healthy controls. MPAs were identified by CD14 monocytes positive for CD41a platelets. The classical (CD14 + + CD16-, CM), the intermediate (CD14 + + CD16+, IM) and the non-classical (CD14 + CD16++, NCM) monocytes, as well as subset specific MPAs, were measured by flow cytometry.

**Results:**

Patients with nephrotic syndrome showed a higher percentage of circulating MPAs as compared with healthy controls (*p* < 0.001). The percentages of MPAs with CM, IM, and NCM were higher than those of healthy controls (*p* = 0.012, *p* < 0.001 and *p* < 0.001, respectively). Circulating MPAs showed correlations with hypoalbuminemia (*r*=-0.85; *p* < 0.001), hypercholesterolemia (*r* = 0.54; *p* < 0.001), fibrinogen (*r* = 0.70; *p* < 0.001) and D-dimer (*r* = 0.37; *p* = 0.003), but not with hypertriglyceridemia in nephrotic syndrome. The AUC for the prediction of hypercoagulability in nephrotic syndrome using MPAs was 0.79 (95% CI 0.68–0.90, *p* < 0.001). The sensitivity of MPAs in predicting hypercoagulability was 0.71, and the specificity was 0.78.

**Conclusion:**

Increased MPAs were correlated with hypercoagulability in nephrotic syndrome. MPAs may serve as a potential biomarker for thrombophilic or hypercoagulable state and provide novel insight into the mechanisms of anticoagulation in nephrotic syndrome.

## Introduction

As knowledge of nephrotic syndrome evolves, hypercoagulability emerges as a central pathological feature and clinical complication. Accumulating evidence has demonstrated a hypercoagulability with high risk of life-threatening thromboembolic complications such as pulmonary embolism, ischemic stroke, and myocardial infarction in nephrotic syndrome [1–5]. Although the underlying causes of the increased risk for thromboembolism are not completely understood, it is well known that platelet hyperactivity plays a fundamental role in thrombosis in nephrotic syndrome. In addition, as high molecular weight platelet binding proteins, elevated plasma levels of von Willebrand factor (vWF) and fibrinogen that contribute to platelet adhesion and aggregation and play an important role in platelet activation, have been described in patients with the nephrotic syndrome [6]. Previous studies have provided evidence indicating that patients with the nephrotic syndrome have platelet hyperaggregability, increased release of active substances, and elevated surface expression of activation-dependent platelet markers [5, 6]. Therefore, coagulation related to platelet activation and aggregation is important process that contributes to prothrombotic state and hypercoagulability in nephrotic syndrome.

Besides releasing granule contents that are procoagulant, activated platelets are prone to bind monocytes, to form monocyte-platelet aggregates (MPAs), which are also conductive to thrombus formation [7, 8]. MPAs are complexes detectable in the circulation and have been proposed to represent a robust biomarker of platelet activation in vivo [7]. Moreover, a study by Michelson et al. [8] has suggested that circulating MPAs are a more sensitive marker of in vivo platelet activation than platelet surface P-selectin. Interaction of platelets with monocytes is considered a crucial pathophysiologic mechanism at the connection of thrombosis and immune inflammation, and contributes to the pro-inflammatory and prothrombotic state [9–11]. Activated platelets secrete many proinflammatory and prothrombotic mediators that participate in inflammatory reaction and hypercoagulable state [9]. Monocytes are a heterogeneous cell population with three dominant subsets according to the expression of surface markers CD14 and CD16, including CD14 + + CD16- (classical monocytes, CM), CD14 + + CD16+ (intermediate monocytes, IM), and CD14 + CD16++ (non-classical monocytes, NCM) subpopulations that exhibit different functional properties [12]. Platelets can bind to three monocyte subsets and to promote monocytes inflammation. Moreover, activated platelets are required to accelerate monocyte-driven inflammation and thrombosis. Several studies have found increased MPAs in patients with diabetes mellitus, unstable angina, acute myocardial infarction, autoimmune disorders, stroke, COVID-19, and in those at risk of thrombosis [13–18]. The level of MPAs reflects the degree of platelet activity thus providing a robust index of thrombosis [7–10]. MPAs are likely to relate to hypercoagulability and thrombosis through the platelet activation, increased cytokine production and expression of cell-adhesion molecules. Therefore, MPAs are surrogate markers of clinical risk of thrombosis [7, 8]. However, the relationship between MPAs and hypercoagulable state in nephrotic syndrome has not been examined. The aim of this study was to investigate the circulating MPAs levels, to evaluate their association with hypercoagulability, and to provide a helpful marker for predicting hypercoagulability risks in nephrotic syndrome. In addition, targeting MPAs effectively reduces MPAs and offers therapeutic potential in preventing thrombosis as shown in several studies [11, 16]. Therefore, understanding the association between MPAs and hypercoagulability may implicate potential targets for intervention.

## Materials and methods

### Study population

The study was approved by the Medical Ethical Committee of First Affiliated Hospital of Harbin Medical University (2023IIT312), and informed consent was obtained from each participant. Thirty-two patients were included in the study. All patients, who aged over 18 years, were newly diagnosed with primary nephrotic syndrome based on clinical manifestations, laboratory tests and kidney biopsy in the department of Nephrology of the First Affiliated Hospital of Harbin Medical University, China. Exclusion criteria included history of secondary nephrotic syndrome, autoimmune diseases, IgA vasculitis nephritis, diabetes mellitus, malignant disorder, hepatitis, liver cirrhosis or abnormal liver function, the acute phase of infection, pregnancy, recent trauma and surgery, use of medications known to affect platelet and coagulation function, and use of statins. All patients were not treated with steroids or immunosuppressants because of newly diagnosed cases. In addition, thirty-two healthy age and sex matched adult volunteers served as healthy controls.

### Clinical data collection

Clinical and laboratory data regarding patients with nephrotic syndrome were documented from electronic medical record database, including age, sex, albumin, alanine aminotransferase, aspartate aminotransferase, total cholesterol, triglyceride, uric acid, serum urea, serum creatinine, 24 h urine protein, leukocyte, hemoglobin, thrombocyte, fibrinogen, and D-dimmer. Estimated glomerular filtration rate (eGFR) was calculated according to the Chronic Kidney Disease Epidemiology Collaboration (CKD-EPI) formula [19] (ml/minute/1.73m2 body surface area). The hypercoagulable state which was the tendency to develop thrombosis, was defined as fibrinogen levels > 3.5 g/L and/or D-dimer levels > 0.55 mg/L FEU.

### Measurement of monocyte-platelet aggregates

Flow cytometric analysis was performed using the flow cytometer(BECKMAN COULTER, USA) and analyzed using FlowJo software (USA). Before clinical treatment peripheral blood samples were obtained in vacuum tubes anticoagulated with 3.2% sodium-citrate after overnight fast. Mouse antihuman monoclonal fluorochrome-conjugated antibodies CD14-FITC (BD Biosciences, USA), CD16-PEcy7 (BD Biosciences, USA), and CD41a- PE (BD Biosciences, USA) were mixed with 50 µl of fresh anticoagulated whole blood in tubes. After incubation for 30 min at room temperature by the manufacturer’s instructions, red blood cells were lysed by 500 µl of lysing solution (BECKMAN, USA) for 15 min, and then cells were washed by adding 500 µl phosphate-buffered saline (PBS) and centrifugation at 1500 rpm for 5 min, followed by dilution in 500µL of PBS and immediate flow cytometric analysis [14]. Whole blood was stained with CD41a-PE to identify platelets, CD14-FITC to identify monocytes and CD16-PEcy7 to identify monocyte subsets. Monocytes were gated according to their characteristic FSC/SSC profiles in peripheral blood mononuclear cells and then identified according to their CD14 surface expression on the mononuclear cells. MPAs were defined as monocytes double positive for CD14 and CD41a, and results were expressed as a percentage of MPAs in monocytes. Next, monocytes were subclassified according to CD14 and CD16 expression into CM, IM and NCM. Subsets were then further analyzed with regard to CD41a expression. The percentage of monocyte subsets in monocytes and the percentage of MPAs with different monocyte subsets in monocytes were calculated. Events (100,000) were collected for each sample.

### Statistical analysis

Continuous data were subject to Shapiro-Wilks test for normality of distribution. Normally distributed data were expressed as mean ± standard deviation (SD). Non-normally distributed continuous data were expressed as median and Q1-Q3 ranges. Comparisons of quantitative variables between two groups were analyzed using the student t test. A Mann–Whitney test was applied as a nonparametric test. Categorical variables were analyzed using Fisher’s exact test. Pearson’s correlation analyses were used to evaluate the associations between MPAs and measured parameters in the patients with nephrotic syndrome. Receiver operator characteristic (ROC) curves were generated to calculate the areas under the ROC curves (AUCs), and to identify the predictive value of MPAs. Differences were considered statistically significant for *P* < 0.05. SPSS version 20.0 (SPSS Inc., Chicago, IL) was used to perform statistical analysis.

## Results

### Clinical characteristics

Baseline characteristics of the study patients were given in Table 1. Thirty-two patients with nephrotic syndrome and thirty-two healthy controls were included in the study.

**Table 1:** The clinical characteristics in all the patients and healthy controls

Characteristics	Healthy controls	Patients with nephrotic syndrome	*p*-value
Number of patients	32	32	
Age (years)	49(38–56)	50(43–56)	0.81
Sex, male, n (%)	19(59.4%)	21(65.6%)	0.80
Albumin (g/L)	45.20 ± 2.76	22.14 ± 3.29	< 0.001
Total cholesterol (mmol/L)	5.57 ± 0.98	8.08 ± 2.48	< 0.001
Triglycerides (mmol/L)	2.21 ± 1.68	2.74 ± 1.38	0.17
Serum urea (mmol/L)	5.10 ± 1.27	5.08 ± 1.73	0.97
Serum creatinine (µmol/L)	70.07 ± 14.41	75.85 ± 19.78	0.19
Serum uric acid (mmol/L)	338.62 ± 98.29	329.28 ± 92.48	0.70
eGFR(ml/min/1.73m2)	104.00 ± 10.36	99.03 ± 18.84	0.20
Leukocyte(×10^9^/L)	6.88 ± 3.04	7.44 ± 2.41	0.43
Hemoglobin (g/dl)	15.16 ± 1.44	13.50 ± 1.87	< 0.001
Thrombocyte (×10^9^/L)	257.85 ± 68.71	267.30 ± 61.89	0.57
Fibrinogen (g/L)	2.64 ± 0.43	5.17 ± 1.39	< 0.001
D-dimer(mg/L FEU)	0.53(0.31–1.58)	1.50(1–2)	< 0.001

### Monocyte subsets in nephrotic syndrome

**Fig. 1:**
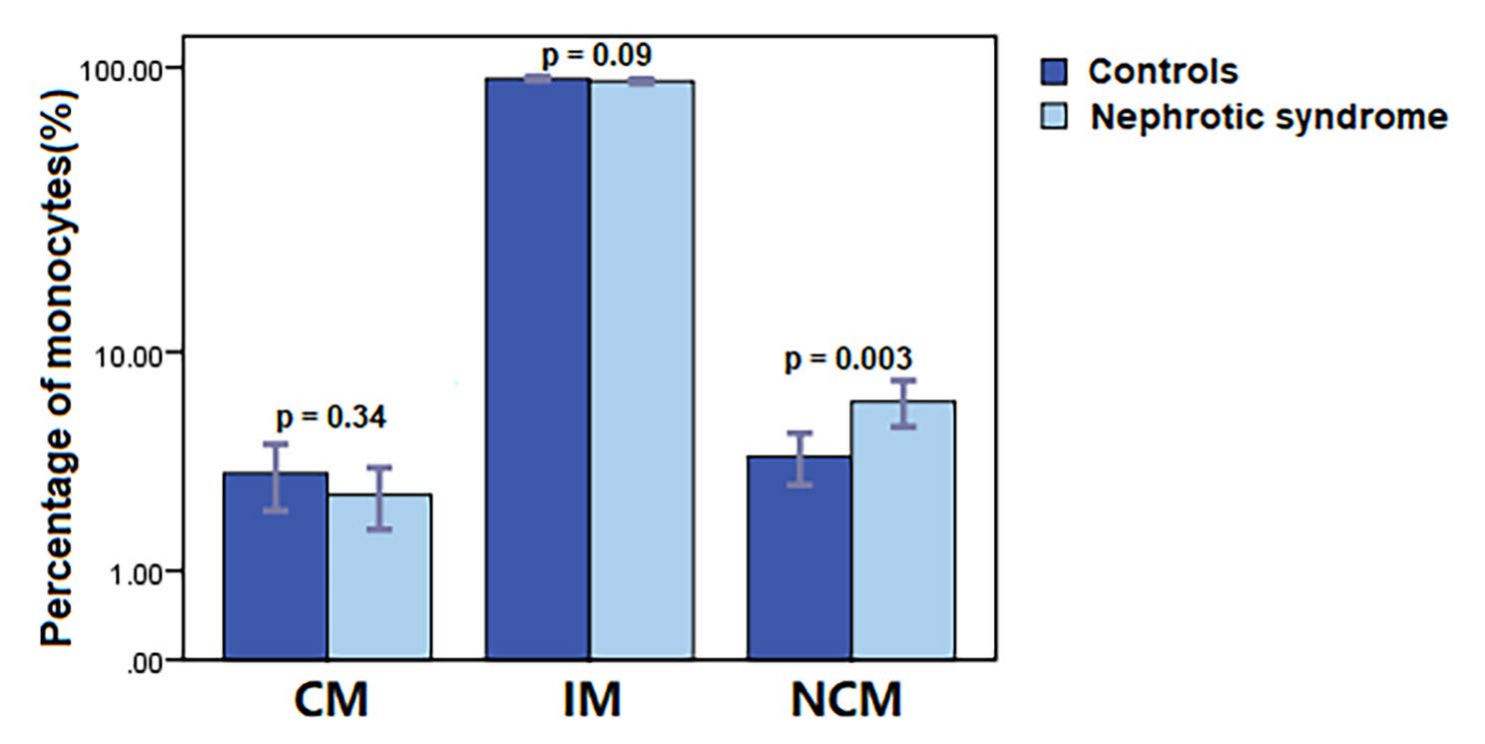
Analysis of the monocyte subsets. Patients with nephrotic syndrome show higher percentage of NCM in comparison to healthy controls (*p* = 0.003)

Monocyte subsets were identified as CMs, IMs, and NCMs by flow cytometry. The percentages of CMs, IMs, and NCMs in patients with nephrotic syndrome were 2.61 ± 2.41%, 89.57 ± 4.60%, and 6.47 ± 3.77% respectively. Patients with nephrotic syndrome showed a significantly higher proportion of NCM as compared with healthy controls (3.87 ± 2.74%, *p* = 0.003, Fig. 1). In contrast, the percentages of CMs and IMs were similar in nephrotic syndrome and healthy controls (3.28 ± 3.07% and 91.49 ± 4.38%).

### The proportion of MPAs in nephrotic syndrome

**Fig. 2:**
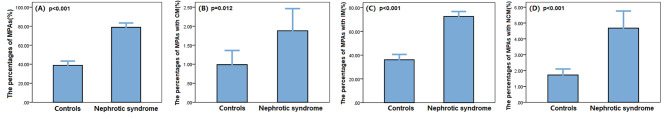
The percentage of MPAs and MPAs with different monocyte subsets in the monocytes in the patients with nephrotic syndrome and healthy controls. (**A**) The percentage of MPAs in the patients with nephrotic syndrome is significantly higher than those of healthy controls (*p* < 0.001). (**B**), (**C**) and (**D**) show the higher percentages of MPAs with CM, IM, and NCM in the patients with nephrotic syndrome in comparison to healthy controls (*p* = 0.012, *p* < 0.001 and *p* < 0.001, respectively)

MPAs formation was a sensitive marker of platelet activation and promoted a pro-thrombotic milieu at the site of platelet activation. The percentage of circulating MPAs in all patients with nephrotic syndrome was significantly higher than those of healthy controls (78.82 ± 12.86% versus 38.83 ± 12.61%, *p* < 0.001). In addition, The percentage of MPAs with different monocyte subsets was analyzed individually. The percentages of MPAs with CM, IM, and NCM (1.88 ± 1.65%, 72.52 ± 11.77%, and 4.68 ± 3.06%, respectively) were significantly higher than those of healthy controls (0.98 ± 1.05%, *p* = 0.012; 36.03 ± 12.80%, *p* < 0.001; and 1.72 ± 1.06%, *p* < 0.001, respectively, Fig. 2).

### Correlation of MPAs with albumin, total cholesterol, triglyceride, fibrinogen and D-dimer in nephrotic syndrome

**Fig. 3:**
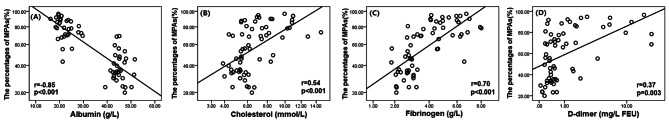
The correlation of MPAs with albumin, total cholesterol, fibrinogen and D-dimer. The percentages of MPAs are correlated with (**A**) hypoalbuminemia (*r*=-0.85, *p* < 0.001), (**B**) hypercholesterolemia (*r* = 0.54, *p* < 0.001), (**C**) fibrinogen (*r* = 0.70, *p* < 0.001) and (**D**) D-dimer in patients with nephrotic syndrome(*r* = 0.37, *p* = 0.003)

Next, associations of circulating MPAs with hypoalbuminemia, hypercholesterolemia, hypertriglyceridemia, fibrinogen and D-dimer were analyzed, to identify correlations of MPAs with hypercoagulability. Circulating MPAs showed significant correlations with hypoalbuminemia (*r*=-0.85, *p* < 0.001), hypercholesterolemia (*r* = 0.54, *p* < 0.001), fibrinogen (*r* = 0.70, *p* < 0.001) and D-dimer (*r* = 0.37, *p* = 0.003, Fig. 3), but not with hypertriglyceridemia in nephrotic syndrome.

### Predictive values of MPAs for hypercoagulability in nephrotic syndrome

**Fig. 4:**
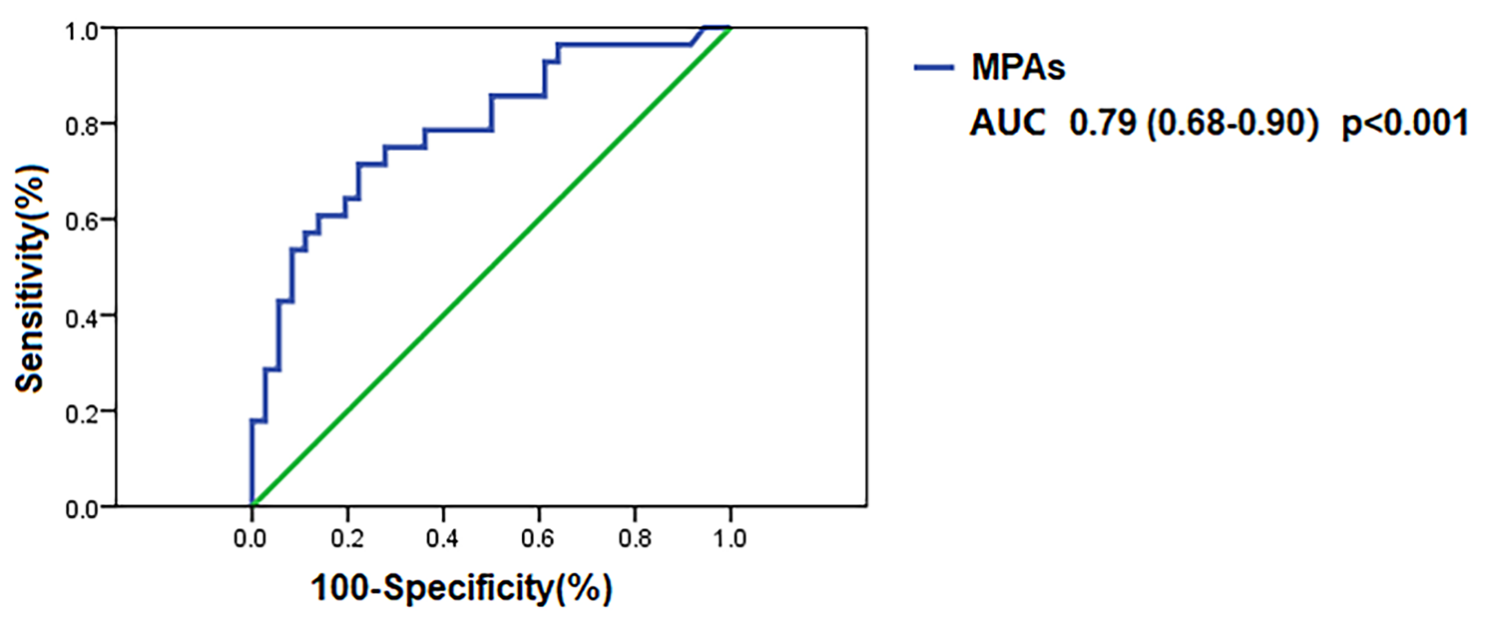
Receiver operating characteristic (ROC) curves of MPAs for predicting hypercoagulability in nephrotic syndrome. AUC indicates area under curve. The number in parentheses of legends indicates 95% confidence of intervals

Subsequently, the areas under the receiver operating characteristic curves (AUC) for the prediction of hypercoagulability in nephrotic syndrome were calculated using MPAs. The AUC for MPAs was 0.79 (95% CI 0.68–0.90, *p* < 0.001, Fig. 4). The Youden index was 0.49 and the cutoff point for MPAs was 68.65%. The sensitivity of MPAs in predicting hypercoagulability was 0.71, and the specificity was 0.78. When the percentage of MPAs was > 68.65%, the risk of hypercoagulability was significantly increased in nephrotic syndrome (OR = 8.75, 95% CI 2.81–27.24, *p* < 0.001).

## Discussion

In the present study the percentage of MPAs was found to be significantly elevated in the patients with nephrotic syndrome compared to healthy controls. MPAs are elevated in multiple thromboinflammatory diseases and correlate with disease severity [13–18]. In peripheral blood, activated platelets with increased surface P-selectin bind to P-selectin glycoprotein ligand 1 (PSGL-1) expressing monocytes to form MPAs [20]. Platelets and monocytes also come in contact through CD40L-CD40 interactions [11, 20]. Additionally, activated platelets adhere to monocytes through bridging molecules such as fibrinogen, which is reported to assist in complex formation, linking Mac-1 on the monocyte surface and integrin αIIbβ3 on platelets [21]. In addition to interaction of platelets with monocytes receptor, platelets can attract monocytes through chemokines and cytokines released from alpha and dense granules. Furthermore, monocytes attached to platelets increase tissue factor expression on their surface, resulting in thrombin-mediated fibrin generation and clot formation [11]. Activated platelets and monocytes tightly interconnected pathogenic mechanisms that profoundly impact on hypercoagulable state in nephrotic syndrome. Previous studies have shown that MPAs, which promote the thrombogenic tendency at the site of platelet activation, are regarded as a sensitive marker of platelet activation and closely correlated with thromboembolic events [13–16, 22]. In the present study, therefore, elevations in MPAs suggested excess activation of platelets and thrombophilic risk in nephrotic syndrome.

Additionally, the binding of platelets and monocytes highlights the integral overlap between inflammation and thrombosis in nephrotic syndrome. In the present study, a significantly higher proportion of NCM was found in nephrotic syndrome as compared with healthy controls. CD16-positive monocytes have been described as potent proinflammatory cells as the proportion of these cells increases in many diseases with an underlying acute or chronic inflammatory stimulus [23]. Furthermore, these cells exhibit a stronger inflammatory answer after activation with various stimuli. Although the pathogenesis of thrombosis in nephrotic syndrome is not completely clear, it is supposed that abnormal activation of platelets in circulation together with inflammatory response has a central role in the formation of thrombosis. In the present study, significantly increased MPAs in peripheral blood of nephrotic syndrome confirmed results of previous studies [1, 5, 6]. Platelets are also known to modulate monocytes secretion of cytokines and chemokines [24, 25]. Previous study has shown that MPAs change the monocyte cytokine profile. The interaction of platelets with monocytes has physiological inflammatory consequences, but also contributes to the propagation of inflammatory damage in disease progression. Recent studies have shown that platelet-induced procoagulant and proinflammatory signaling in monocytes are linked, amplifying inflammation and hypercoagulability in disease [26–28]. Therefore, intense proinflammatory cytokines and procoagulant factors production may contribute to hyperinflammation and hypercoagulability in the nephrotic syndrome. Accumulating evidences have indicated that increased interaction of platelets and monocytes and circulating MPAs suggest a propensity of thrombosis in disease [11, 26–28]. Thus, we speculate that MPAs may participate in the thrombophilic or hypercoagulable process in nephrotic syndrome. However, new studies are still necessary to determine how MPAs amplify inflammation and hypercoagulability in nephrotic syndrome.

In this study, MPAs were closely associated with hypoproteinemia, hypercholesterolemia, increased coagulation biomarker fibrinogen and D-dimer, indicating a potentially higher platelet activation and thromboembolic risk in patients with the nephrotic syndrome. Our results indicate positive correlation between the presence of MPAs and susceptibility to thrombosis. Previous studies have shown that platelet activity usually inversely correlate with hypoalbuminemia which reflected severity of nephrotic syndrome, and it is potentially reversible by adding albumin in vitro or following albumin infusions [29]. Risks for thromboembolic events in nephrotic syndrome increase with severe hypoalbuminemia. Subsequently, coagulation activation in nephrotic syndrome is accompanied by increased levels of fibrinogen which are the hepatic synthetic response to the hypoalbuminemia. D-dimer, which is a byproduct of fibrin degradation, is used in several clinical settings, such as in predicting venous thrombosis, ischemic stroke and cardioembolic stroke, and regarded as markers linked with thrombophilic or hypercoagulable state in clinical practice [30]. Additionally, previous studies have indicated that hypercholesterolemia also promotes platelet adhesiveness and hyperactivity and relates to hypercoagulability in nephrotic syndrome [31]. Therefore, the significant correlation of MPAs with hypoalbuminemia, hypercholesterolemia, fibrinogen and D-dimer supports its role in hypercoagulable state and thrombosis in nephrotic syndrome. In this study, MPAs in nephrotic syndrome patients were significantly higher than those in healthy controls, and correlated with indexes related to hypercoagulable state, suggesting that MPAs is involved in the development of hypercoagulability in nephrotic syndrome. Moreover, with MPAs > 68.65%, patients with nephrotic syndrome were more prone to present hypercoagulable state or thrombosis. Our results indicate a heightened crosstalk between platelets and monocytes in the setting of nephrotic syndrome, and provide novel insight to the heightened thromboembolic or hypercoagulable risk in nephrotic syndrome.

The study has several limitations. Firstly, cross-sectional study and the relatively small sample size in this study prevent us from evaluating the impact of MPAs on clinical outcomes. Larger cohort studies in the future are warranted for in-depth analysis of the association between MPAs and prognosis in nephrotic syndrome. Especially, we would like to further explore the associations of the MPAs with duration of the disease and the extent of proteinuria with the remission of the disease. Additionally, the functional implications of MPAs will require further study, because it remains unclear whether formation of MPAs is the simply reflection of the underlying thrombophilic and hypercoagulable state in nephrotic syndrome or whether they directly contribute to the pathophysiology of the disease development. Finally, the dynamic analysis of MPAs should be further evaluated in the course of nephrotic syndrome in the future.

## Conclusion

In conclusion, this study demonstrated that increased MPAs were correlated with hypercoagulability in nephrotic syndrome. The increased crosstalk between monocytes and platelets might contribute to thrombophilic risk. MPAs *may* serve as a potential biomarker for thrombophilic or hypercoagulable state in nephrotic syndrome. Although the further recognition of the mechanism of MPAs in thrombophilic or hypercoagulable state is needed, it may provide novel insight into the mechanisms of anticoagulation in nephrotic syndrome.

## Data Availability

No datasets were generated or analysed during the current study.

## Abbreviations

MPAs: Monocyte platelet aggregates
vWF: von Willebrand factor
eGFR: Estimated glomerular filtration rate
CKD-EPI: Chronic Kidney Disease Epidemiology Collaboration
CM: Classical monocytes
IM: Intermediate monocytes
NCM: Non-classical monocytes
SD: Standard deviation
AUC: The areas under the receiver operating characteristic curves
ROC: Receiver operator characteristic
